# The associations between smart device use and psychological distress among secondary and high school students in Kuwait

**DOI:** 10.1371/journal.pone.0251479

**Published:** 2021-06-15

**Authors:** Ali Jasem Buabbas, Huda Hasan, Mohammad Abbas Buabbas

**Affiliations:** 1 Department of Community Medicine and Behavioural Sciences, Faculty of Medicine, Kuwait University, Kuwait; 2 Department of Psychology, Faculty of Social Sciences, Kuwait University, Kuwait; 3 Department of Physical Education, College of Basic Education, The Public Authority for Applied Education and Training, Kuwait; Semmelweis University, HUNGARY

## Abstract

**Background:**

Smart devices (SDs) are widely used among adolescents. Numerous studies have recommended further research on this topic to find out the prevalence of SD overuse among school students and to what extent this is associated with psychological distress. The present study aimed to investigate the pattern of SD use among secondary and high school students in the state of Kuwait, as well as the possible associations with psychological problems, weight, physical activity, and school performance.

**Materials and methods:**

The correlational study design aimed to survey students from public schools by using a questionnaire and valid instruments, which included: Smartphone Addiction Scale–Short Version (SAS-SV) and Stress, Anxiety, and Depression Scale–(DASS-21). Pearson’s correlation coefficient, *t*-tests, one-way ANOVA were applied to find associations or significant differences between the categorical variables, in which p < 0.05 was considered statistically significant.

**Results:**

The study included 1,993 students from secondary schools (48.9%) and high schools (51.1%), of which 47.5% were male and 52.5% were female. There were significant statistical differences in the pattern of use of SDs, addiction to SD use, stress, anxiety, and depression according to gender, school type, school performance, and sport engagement. In addition, there were positive correlations between students’ addiction to SD use and stress, anxiety, and depression.

**Conclusions:**

The findings suggest that excessive SD use is associated with addiction among secondary and high school students. In addition, levels of stress, anxiety, and depression differ according to the number of hours of SD use. Strategies should be developed at the community and school levels to avoid the overuse of SDs among school-aged students.

## Introduction

Currently, in Kuwait, the widespread use of smart devices (SDs), such as smartphones (SPs) and tablets, particularly among adolescents, is apparent, with easy internet access making users constantly connected to the rest of the world. SDs are considered a basic tool for spending time, whether utilising their various applications for productivity or entertainment. In the US, Pew Research Center reported that nearly 95% of teens had access to SPs, and many of them had concerns about overusing SPs [1]. A study in South Korea found that SP use was more common among the age group of 10–20 years old than the age group of 20–30 years old [2]. The internet service offered by SDs fulfils users’ need for online communication (e.g. social media or playing games) [3]; hence, the most frequent internet users are adolescents and young adults [4]. Previous studies have reported that mental health is influenced by patterns of internet use [3, 5]. It has been found that adolescents are the most uncritically receptive of new technology and easily adapt to it, and they are willing to imitate others in using new applications; hence, they are more vulnerable to the adverse effects of SP overuse [4, 6].

### SD overuse and associated factors

According to previous studies in this context, several factors are associated with SD overuse. In Korea, a study found that SP addiction among young children was associated with problematic behaviour and altered emotional intelligence [7]. A systematic review revealed the causal relationship between SP addiction and psychological problems, including depression, anxiety, and stress [8]. Similarly, this association was found among adolescent online gamers and was accompanied by internet gaming disorders [9, 10, 11]. Previous studies have shown that the everyday functioning, interpersonal relationships, and emotional well-being of individuals are negatively affected by problematic internet use [12, 13].

The findings of a systematic review suggested that problematic internet use among adolescents / young adults has more-significant relationships with individual factors than with contextual and activity-related factors [14]. This could indicate that an individual’s core characteristics (e.g. genetics), predisposing factors (e.g. early childhood experiences), and several mediators and moderators (e.g. coping styles and cognitive-related biases) are all factors that contribute to internet addiction [11].

### SD use among adolescents

Adolescents enjoy technology and may overuse it without awareness of the potential negative consequences, as they have less-developed self-control compared to adults [7, 15]. Therefore, addiction to SD use among adolescents is becoming a major issue in society [4].

In addition, significant differences have been reported in the pattern of SD use according to gender, which is considered an important variable that requires comparative study [16, 17]. This difference might be due to the different interests of males and females. For instance, males may have more interest in internet games, while females may be more interested in social media [17].

### Distress associated with SD overuse

Regardless of the advantages of SDs, detrimental effects are becoming apparent in society. The excessive use of SPs among adolescents can create isolation and loneliness and trap them in a triangle of associated feelings: depression, anxiety, and stress [18]. A longitudinal study of 2,286 European adolescents found an association between the magnitude of internet use and poor mental health, based on a four-month follow-up using valid scales of depression, stress, and anxiety [5].

Depending on the quality of interactions and individual factors, social media can have both detrimental and beneficial effects. A systematic review of 70 studies found that social media use was correlated with depression, anxiety, and low scores on measures of well-being [14, 19]. Previous studies have reported the negative consequences of SD overuse/addiction, including reduced performance (particularly school performance) [6], more-sedentary behaviour [20], obesity [21], impaired parent–teen relationships, and altered personalities [21, 22]. It is particularly important to consider these effects because adolescences are in a stage of not only physical growth but also behavioural and intellectual growth.

Previous studies have recommended further research among adolescents in diverse populations and cultures regarding the prevalence of SD overuse, its risk factors and its potential detrimental effects such as psychological distress, educational performance, physical activity, and overweight [18, 23, 24]. Despite the importance of this topic, there is a lack of relevant published research among adolescents in Kuwait. Therefore, this study aimed to investigate these issues in the context of secondary and high schools in the state of Kuwait.

The research hypotheses included, 1) SD overuse/addiction is prevalent among secondary school students in Kuwait; 2) SD overuse/addiction is significantly and positively associated with psychological disorders (stress, anxiety, and depression) and overweight; and 3) There are significant differences in the associations between SD overuse/addiction and psychological disorders (stress, anxiety, and depression) according to gender, school type, school performance, and sport engagement.

## Materials and methods

### Study design, participants, and research setting

This correlational study was based on a survey of students from public secondary and high schools. The public schools in Kuwait are separated based on educational level and gender. The data was collected from 2,146 students from public schools (secondary and high schools) between year 7 and year 12. There were 1,993 useable questionnaires, so the response rate was approximately 93%. The participants were Kuwaitis and non-Kuwaitis of both genders (male = 947, female = 1,046) and from all six educational regions in Kuwait (Asimah (Capital), Hawally, Farwaniya, Jahra, Ahmadi, and Mubarak Alkabeer). The participants’ ages ranged between 11 and 21 years (M = 15.28, SD = 1.71). Their heights ranged between 62 and 194 cm (M = 161.80, SD = 10.56), while their weights ranged between 31 and 174 kg (M = 66.18, SD = 20.74). In total, 92% of the participants used SDs. Table 1 shows the distribution of the demographic data of the sample.

**Table 1:** Demographic data of the participants.

Variable	n	%
**Educational Region:** Asimah Hawally Mubarak Alkabeer Farwaniya Jahra Ahmadi	326 347 333 313 353 321	16.4 17.4 16.7 15.7 17.7 16.1
**School type:** Secondary school High school	974 1,019	48.9 51.1
**Gender:** Male Female	947 1,046	47.5 52.5
**Nationality:** Kuwaiti Non-Kuwaiti	1,726 267	86.6 13.4
**Educational Level:** 7 8 9 10 11 12	71 201 703 76 358 584	3.6 10.1 35.3 3.8 18.0 29.3
**Last overall grade:** F (<60) D (60–69) C (70–79) B (80–89) A (90–100)	66 195 540 687 477	3.3 9.8 27.1 34.5 23.9

* Missing data was excluded from analysis.

### Research instrument

The demographic data gathered from the participants included age, gender, nationality, school type, educational level, last overall grade, educational region, height, weight, average total hours of SD use per day, and average time spent on SDs per session. The data was collected using two scales:

#### The Smartphone Addiction Scale–Short Version (SAS-SV)

This short version of the smartphone addiction scale contains 10 items [24], which was measured on a four-point Likert scale (4 = strongly agree, 3 = agree, 2 = disagree, 1 = strongly disagree). The total score for the SAS-SV ranges between 10 and 40. A high score meant that the student had a high addiction to SP use. The students were asked to select the appropriate response for each item of the scale, which included: (1) “Missing planned work due to smartphone use” and (2) “Won’t be able to stand not having a smartphone”. The reliability of the scale was measured by the Cronbach’s alpha value, which was high (.814).

#### The Stress, Anxiety, and Depression Scale–(DASS-21)

This scale contains 21 items, with each subscale (stress, anxiety, and depression) measured by seven items [25]. The participants’ responses are assessed on a four-point Likert scale (0 = never applies to me, 1 = sometimes applies to me, 2 = often applies to me, 3 = always applies to me), and the total score for the scale ranges between 0 and 21. A high score meant that the student had a strong psychological disorder. The students were asked to select the appropriate answers for items such as “I found it difficult to minimise the use of smart devices” (stress), “I was aware of dryness of my mouth” (anxiety), and “I couldn’t seem to experience any positive feeling at all” (depression). The reliability of each subscale was assessed using the Cronbach’s alpha value, all of which were acceptable (stress = .73, anxiety = .73, depression = .75).

The questionnaire was translated from English into Arabic [S1 and S2 Files]. Back translation was performed on the scales to ensure accuracy. The translation was performed by the Translation Office at the Faculty of Medicine of Kuwait University.

Prior to the official distribution of the Arabic version of the questionnaire, it was tested with five students to ensure the suitability and readability of the questions. No items were considered ambiguous, and no language concerns were raised by the students.

### Data collection in the research setting

A two-stage cluster sampling technique was used in this research. In each educational region, for each gender, two secondary schools and two high schools were randomly selected. This produced 48 schools, each of which had 96 classes, and the average number of students in each class was 23.

The participants were approached in their classes by the first and third authors and were asked to complete the questionnaire, which was distributed by hand to the students. The researchers waited to collect the completed questionnaires.

### Eliciting ethical approval

Approval for the study was elicited from the Research Ethics Committee at the Kuwait Ministry of Health (reference number: 885, 2018). Accordingly, written informed consent was obtained from one parent of each of the participants, and the participants were informed that they were free to withdraw from the study at any time.

### Statistical analysis

The data management, analysis, and graphical presentation were achieved using the computer software Statistical Package for the Social Sciences (SPSS), version 25.0 (IBM Corp.). The body mass index (BMI) of the students was calculated using the following formula: BMI = weight / (height)^2^ (Centers for Disease Control and Prevention (CDC), 2020).

The descriptive statistics are presented as numbers and percentages for the sociodemographic data. The pattern of SD use was determined by the number of hours of SD use per day and the average time spent on SDs per session, accordingly, participants were divided into three types: overuse, moderate use, and less use. One-way analysis of variance and Scheffe tests were used to test the significant differences in addiction, depression, anxiety, and stress, according to the average total hours of SD use per day and per session. Pearson’s correlation coefficient was used to test the strength and direction of the associations between the study variables. In addition, *t*-tests were used to compare the means of the variables according to gender, school type, and sport engagement. Moreover, one-way analysis of variance (ANOVA) and Scheffe tests were used to compare the means of the variables according to the time spent on SDs per day and per session, as well as academic performance level. The effect size (η^2^ and Cohen’s *d*) was tested for the independent variables’ means. Two-tailed analysis were conducted, and P≤ 0.05 was considered significant.

## Results

To find out the prevalence of SD addiction, the SD use was divided into three types: overuse, moderate use, and less use. The pattern of SD use was determined by the number of hours of SD use per day and the average time spent on SDs per session.

The results show that most of the students (1,287, 64.6%) used SDs for >4 hours per day, which was categorised as overuse, as per the Canadian Paediatrics Society [26], while 500 (25.1%) of the students used SDs for 2–4 hours (moderate use) and 197 students (9.9%) used SDs for <2 hours (less use).

ANOVA and Scheffe tests were used to test for differences in addiction, stress, anxiety, and depression according to the average total hours of SD use per day. There were significant differences according to the total hours of SD use per day (p<0.001). Students who used SDs for ˃4 hours per day scored higher on the addiction, stress, anxiety, and depression scales. The effect size (η^2^) was only high for the addiction variable; it was weak for the other variables and ranged between .008 and .025 (see Table 2).

**Table 2:** Means, SDs, and ANOVA for the variables according to the total hours of SD use per day.

Variables	M	SD	*F* (2, 1979)	η^2^
**Addiction:** Less than 2 hours 2–4 hours More than 4 hours	13.70 15.61 19.94	5.60 5.13 5.77	178.99***	.153↑
**Stress:** Less than 2 hours 2–4 hours More than 4 hours	5.73 6.47 7.74	4.33 4.17 4.70	25.70***	.025↓
**Anxiety:** Less than 2 hours 2–4 hours More than 4 hours	4.41 4.62 5.32	4.19 3.97 4.33	7.53***	.008↓
**Depression:** Less than 2 hours 2–4 hours More than 4 hours	4.51 4.95 5.87	4.07 4.01 4.49	13.78***	.014↓

*** p < .001

Regarding the students’ hours of SD use per session, the results show that 1,089 (54.6%) of the students used SDs for >2 hours per session, which is considered overuse; 554 (27.8%) of the students used SDs for 1–2 hours, which is considered moderate use; and 339 (17%) of the students used SDs for <1 hour per session, which is considered less use.

The results of the ANOVA and Scheffe tests show that students who spent >2 hours per session using SDs scored higher on the addiction scale compared to other students (p<0.001). Similarly, students who spent >2 hours using SDs per session scored higher on the stress and depression scales compared to those who spent <1 hour on SDs per session (p<0.05), but no significant differences were found on the anxiety scale according to the number of hours spent using SDs per session. The effect size (η^2^) was weak for all of the variables and ranged between .000 and .057 (see Table 3).

**Table 3:** Means, SDs, and ANOVA for the variables according to the total hours of SD use per session.

Variables	M	SD	*F* (2, 1979)	η^2^
**Addiction:** Less than 1 hr Between (1hr-2hr) More than 2hr	15.96 17.14 19.50	5.72 5.86 6.00	59.36***	.057↓
**Stress:** Less than 1 hr Between (1hr-2hr) More than 2hr	6.62 7.25 7.38	4.35 4.45 4.73	3.59*	.004↓
**Anxiety:** Less than 1 hr Between (1hr-2hr) More than 2hr	4.94 5.02 5.10	4.17 4.19 4.28	.22 (NS)	
**Depression:** Less than 1 hr Between (1hr-2hr) More than 2hr	4.92 5.66 5.66	4.07 4.21 4.50	3.79*	.004↓

* p < .05

*** p < .001. Note: Missing data was excluded from the analysis.

In addition, Pearson’s correlation coefficient was used to test the strength and direction of the relationships between the variables. Table 4 shows that students’ addiction to SD use was significantly correlated with feelings of stress, anxiety, and depression (p<0.01). Moreover, the results show that there were weak correlations between BMI and stress, anxiety, and depression.

**Table 4:** Correlations between the variables (n = 1993).

Variables	1	2	3	4
**1- Addiction**	-			
**2- Stress**	.42**	-		
**3- Anxiety**	.29**	.67**	-	
**4- Depression**	.32**	.65**	.63**	-
**5- BMI**	.06	.09**	.11**	.08*

* p < .05

** p < .01

*T*-tests were used to compare the values for SD addiction, stress, anxiety, and depression according to gender, school type, and educational performance. The results show that female students scored higher on SD addiction, stress, anxiety, and depression compared to male students (Table 5). The effect size (Cohen’s *d*) was weak for all variables, and it ranged between .028 and .118.

**Table 5:** *T*-tests comparing males (n = 947) and females (n = 1,046) in the study variables (df = 1991).

Variables	M	SD	*t*-value	Cohen’s *d*
**Addiction:** Males Females	17.16 19.22	6.03 5.97	7.64***	.028↓
**Stress:** Males Females	5.56 8.71	3.99 4.59	16.31***	.118↑
**Anxiety:** Males Females	3.82 6.17	3.59 4.47	12.84***	.077→
**Depression:** Males Females	4.55 6.37	3.90 4.56	9.55***	.044↓

*** p < .001

In addition, the results showed that the average scores for SD addiction, stress, anxiety, and depression were higher among high school students compared to secondary school students. However, the differences were weak because the effect size (Cohen’s *d*) was weak for all of the variables, and it ranged between .004 and .010 (Table 6).

**Table 6:** *T*-tests comparing secondary school students (n = 974) and high school students (n = 1,019) in the study variables (df = 1991).

Variables	M	SD	*t*-value	Cohen’s *d*
**Addiction:** Secondary School High School	17.85 18.61	6.52 5.61	2.79**	.004↓
**Stress:** Secondary School High School	6.81 7.59	4.71 4.46	3.82***	.007↓
**Anxiety:** Secondary School High School	4.75 5.34	4.37 4.10	3.06**	.005↓
**Depression:** Secondary School High School	5.06 5.93	4.41 4.26	4.50***	.010↓

** p < .01

*** p < .001

Furthermore, Table 7 presents the ANOVA and Scheffe test results in relation to school performance. It shows significant differences in students’ SD addiction according to their school performance. Students with better school performance were less addicted to SD use. In addition, students with better school performance scored higher in stress compared to students with lower performance; on the other hand, lower-performing students scored higher in depression compared to students with better performance.

**Table 7:** Means, SDs, and ANOVA for the variables according to school performance level.

Variables	M	SD	*F* (4, 1960)	η^2^
**Addiction:** F (<60) D (60–69) C (70–79) B (80–89) A (90–100)	19.18 19.20 18.22 18.45 17.31	6.20 6.02 6.32 5.85 6.06	4.64***	.009↓
**Stress:** F (<60) D (60–69) C (70–79) B (80–89) A (90–100)	7.01 7.12 7.04 6.98 7.80	4.81 4.89 4.77 4.44 4.41	2.66*	.005↓
**Anxiety:** F (<60) D (60–69) C (70–79) B (80–89) A (90–100)	5.65 5.29 5.05 4.82 5.25	4.83 4.38 4.33 4.11 4.18	1.24 (NS)	
**Depression:** F (<60) D (60–69) C (70–79) B (80–89) A (90–100)	6.84 6.24 5.36 5.21 5.64	4.65 4.24 4.41 4.31 4.32	3.98**	.008↓

*p < .05

**p < .01

***p < .001, F (<60), D (60–69), C (70–79), B (80–89), A (90–100).

On the other hand, no significant differences were found in students’ levels of anxiety according to their school performance. The effect size (η^2^) was weak for all of the variables, and it ranged between .003 and .009.

In addition, there were significant differences between physically active and inactive students in terms of their addiction to SD use and their levels of stress, anxiety, and depression. The results of the *t*-tests indicate that students who did not regularly practise sport scored higher in addiction, stress, anxiety, and depression compared to those who practised sport on a regular basis, but the effect size (Cohen’s *d*) was weak for all of the variables, and it ranged between .002 and .032 (Table 8).

**Table 8:** *T*-tests comparing physically inactive students (n = 1,194) and active students (n = 788) in the study variables (df = 1980).

Variables	M	SD	*t*-value	Cohen’s *d*
**Addiction:** Physically Inactive Physically Active	19.13 16.90	5.93 6.00	8.14***	.032↓
**Stress:** Physically Inactive Physically Active	7.46 6.86	4.58 4.58	2.82**	.004↓
**Anxiety:** Physically Inactive Physically Active	5.23 4.80	4.28 4.14	2.21*	.002↓
**Depression:** Physically Inactive Physically Active	5.71 5.22	4.43 4.22	2.44*	.003↓

* p < .05

** p < .01

*** p < .001

## Discussion

In this study, more than 50% of the sample reported using SDs for more than four hours per day, which could be an indication of overuse. The findings suggest that students’ overuse of SDs could be associated with addiction, stress, anxiety, and depression. Furthermore, there were significant differences in SD addiction, stress, anxiety, and depression according to gender, school type, school performance, and sedentary behaviour. Based on the investigation, the developed hypotheses were supported by the findings of this study.

### Hours of SD use and associated psychological distress

The findings suggest in this study that students who overused SDs (˃4 hours per day) were more addicted to SD use and had higher scores in stress, anxiety, and depression. In addition, students who spent ˃1 hour on SDs per session were more addicted to SD use and had higher scores in stress, and depression.

These findings are consistent with previous studies that found that students are the group most likely to adopt new technologies and become addicted to their use, which is correlated with depression and anxiety [27, 28, 29]. Moreover, the findings of the present study show that the high school students had more-addictive tendencies towards SD use than the secondary school students did. This could be due to the fact that high school students have more friendships and more spare time, so they try to stay connected with others through social media or entertainment applications. Another study found that adolescents are more susceptible than adults are to SD addiction, specifically regarding internet use, and this has a significant impact on adolescents’ mental health [4]. This could be elucidated through considering ‘digital natives’ vs. ‘digital immigrants’, in which the first are the generation who were born in the digital era and are used to using SDs, while others have to learn how to use these devices, so they might use them less.

Additionally, the female students in this study had more-addictive tendencies towards SD use than the male students did, which is consistent with previous studies that found that female students experienced greater levels of addictive SD use [4, 24, 28, 29]. It seems that females are more sociable than males are on social media applications, as they might use them as a platform for the free expression of their thoughts and opinions online. It has been reported that this difference in the pattern of use of social media platforms between the genders can be explained by biological, psychosocial, and sociocultural reasons [30, 31]. However, other studies did not find any differences according to gender in this regard [32].

### SD use and physical activity

Adolescents are typically full of energy and tend to be active; therefore, engaging in less physical activity and exercise can lower their moods, which could explain their development of psychological problems through SD addiction. A survey of Iranian university students found that their mental health improved with reduced mobile addiction [33].

The present study’s findings reveal that the students who practised sport on a regular basis were less addicted to SD use and had lower levels of stress, anxiety, and depression. This is due to the influence of exercise on the mind, as well as on the individual, as those who spend time practising sports have less time for SD use. In China, physical exercise has been used to improve self-control and decrease the dependence of university students on SP use [34].

### SD use and school performance

Furthermore, the students with better school performance were less addicted to SD use. This could be because the overuse of SDs can distract students, decrease their concentration in general, and make them tired, which could affect their school performance. A similar result was found among Korean and US adolescents, where poor school performance was associated with higher SP use [6, 27]. In Lebanon, a study was conducted with university students. It concluded that SP addiction was associated with perceived stress but not school performance [35]. A US study found that the high use of mobile phones by students was positively associated with anxiety and negatively associated with academic performance, as well as positively associated with life satisfaction [36]. By contrast, a UK study found that perceived depression, anxiety, and stress were insignificantly associated with SP addiction [18], explaining that the personality of the individual and their aim in using an SP are behind these psychological problems, not SP overuse.

### SD use and weight

Surprisingly, it was found that the students’ patterns of SD use and levels of stress, anxiety, and depression had weak significant associations with their weight (BMI) for both genders. Although there were weak correlations between weight and other variables for the male students, these were considered insignificant based on the large sample size. Some studies have related adolescent students’ obesity to SD overuse and the subsequent sedentary lifestyle [37, 38, 39, 40].

### Strengths and limitations

A key strength of this study was its large sample size, which involved two levels of education and focused on the target group of adolescents. In addition, the data collection was performed by the first and third authors, who had skills in conducting this process in a professional way, which aided in attaining a high response rate (93%). However, there were limitations in this study: 1) the results of the study cannot be generalised to all school students or adolescents because the survey did not include students of all ages or sociodemographic backgrounds, hence, future research could be involved all ages groups of a population to enhance the generalisability of the findings; 2) the cut-off points of the SDs overuse were not available for the population included in the study, so, determining the cut-off points of the SDs overuse for Kuwait population is recommended in future research; 3) although the excessive use of SDs can lead to addictive tendencies, which in turn can increase levels of stress, anxiety, and depression, in the age of adolescence, these psychological problems are naturally expected and might not be directly associated with SD addiction, as SD addiction is not a universally accepted construct, therefore, the findings of this study could have been enhanced and supported through the use of diagnostic interviews, and further study could be involved ana older age group, which is the youth; and 5) the data was collected from several schools and the students were nested within the schools, so the results may have been affected by the data collection style, as the students were not surveyed independently. A longitudinal study is recommended to confirm the findings.

## Conclusion

This study concluded that SD overuse can influence psychological distress levels among secondary and high school students in Kuwait. The findings suggest that excessive SD use is associated with addiction, as the results indicate that the more hours students use SDs (˃4 hours per day and ˃2 hours per session), the more they become addicted to using SDs, so the greater their levels of stress, anxiety, and depression will be. In addition, the more students use SDs, the more they become inactive and engage in sedentary behaviour.

Thus, it is advisable for students not to exceed a moderate number of hours of SD use per day and per session to avoid detrimental impacts on their mental health, as recommended by the Canadian Paediatrics Society [26]. Furthermore, regularly practising sports can have a positive impact on students in avoiding the overuse of SDs, making students less likely to become addicted to SD use and protecting them from the associated psychological problems.

Overall, it is crucial to make parents, school students, and health professionals aware of the harmful consequences of SD overuse, together with suggesting positive and effective programmes and strategies to create better and healthier lifestyles for children.

## Supporting information

S1 File A questionnaire–Arabic version. (DOCX)

S2 File A questionnaire–English version. (DOCX)

S1 Raw data (SAV)

## References

[1] Schaeffer K. Most U.S. teens who use cell phones do it to pass time, connect with others, learn new things. Pew Research Center. 2019 August-[cited 2020 April 2] Available from: https://www.pewresearch.org/fact-tank/2019/08/23/most-u-s-teens-who-use-cellphones-do-it-to-pass-time-connect-with-others-learn-new-things/

[2] Cha SS, Seo BK. Smartphone use and smartphone addiction in middle school students in Korea: Prevalence, social networking service, and game use. Health Psychology Open. 2018; 5(1):2055102918755046. doi: 10.1177/2055102918755046 29435355 PMC5802650

[3] Kim DI, Lee YH, Lee JY, Kim MC, Keum CM, et al. New Patterns in Media Addiction: Is Smartphone a Substitute or a Complement to the Internet? The Korean Journal of Youth Counselling. 2012; 20(1): 71–88

[4] Kim D, Lee Y, Lee J, Nam JK, Chung Y. Development of Korean Smartphone addiction proneness scale for youth. PLoS One. 2014;9(5):e97920. doi: 10.1371/journal.pone.0097920 24848006 PMC4029762

[5] Hökby S, Hadlaczky G, Westerlund J, et al. Are Mental Health Effects of Internet Use Attributable to the Web-Based Content or Perceived Consequences of Usage? A Longitudinal Study of European Adolescents. JMIR Ment Health. 2016;3(3):e31. doi: 10.2196/mental.5925 27417665 PMC4963606

[6] Kim MH, Min S, Ahn JS, An C, Lee J. Association between high adolescent smartphone use and academic impairment, conflicts with family members or friends, and suicide attempts. PLoS ONE. 2019; 14(7): e0219831. doi: 10.1371/journal.pone.0219831 31306455 PMC6629152

[7] Seu K, Lee J. Influence of smartphones addiction proneness of young children on problematics behaviors and emotional intelligence: Mediating self-assessment effects of parents using smartphones. Computers in Human Behavior. 2017; 66: 303–311. 10.1016/j.chb.2016.09.063

[8] Elhai JD, Dvorak RD, Levine JC, Hall BJ. Problematic smartphone use: A conceptual overview and systematic review of relations with anxiety and depression psychopathology. J Affective Disorders. 2016;2(207):251–259. doi: 10.1016/j.jad.2016.08.030 27736736

[9] Adams BL, Stavropoulos V, Burleigh TL, Liew LW, Beard CL, Griffiths MD. Internet gaming disorder behaviors in emergent adulthood: A pilot study examining the interplay between anxiety and family cohesion. International Journal of Mental Health and Addiction. 2018; 1.

[10] Burleigh TL, Stavropoulos V, Liew LW, Adams BL, Griffiths MD. Depression, internet gaming disorder, and the moderating effect of the gamer-avatar relationship: An exploratory longitudinal study. International Journal of Mental Health and Addiction. 2018;16 (1):102–124, doi: 10.1007/s11469-017-9806-3

[11] Scerri M, Anderson A, Stavropoulos V, Hu E. Need fulfilment and internet gaming disorder: A preliminary integrative model. Addict Behav Rep. 2018;9:100144. doi: 10.1016/j.abrep.2018.100144 31193898 PMC6543453

[12] Akin A. The relationships between Internet addiction, subjective vitality, and subjective happiness. CyberPsychology, Behavior, and Social Networking. 2012; 15: 404–410. doi: 10.1089/cyber.2011.0609 22823517

[13] Anderson E, Steen E, Stavropoulos V. Internet use and Problematic Internet Use: a systematic review of longitudinal research trends in adolescence and emergent adulthood. International Journal of Adolescence and Youth. 2017;22:4: 430–454, doi: 10.1080/02673843.2016.1227716

[14] Seabrook EM, Kern ML, Rickard NS. Social networking sites, depression, and anxiety: A systematic review. JMIR Ment Health. 2016;3(4):e50. doi: 10.2196/mental.5842 27881357 PMC5143470

[15] Shan Z, Deng G, Li J, Li Y, Zhang Y, Zhao Q. Correlational Analysis of neck/shoulder Pain and Low Back Pain with the Use of Digital Products, Physical Activity and Psychological Status among Adolescents in Shanghai. PLoS ONE. 2013; 8(10): e78109. doi: 10.1371/journal.pone.0078109 24147114 PMC3795657

[16] Lee SY, Lee D, Nam CR, Kim DY, Park S, Kwon JG, et al. Distinct patterns of Internet and smartphone-related problems among adolescents by gender: Latent class analysis. Journal of behavioral addictions. 2018; 7(2): 454–465. doi: 10.1556/2006.7.2018.28 29788762 PMC6174601

[17] Taywade A, Khubalkar R. Gender differences in smartphone usage patterns of adolescents. The International Journal of Indian Psychology. 2019; 7:509–515. doi: 10.25215/0704.060

[18] Harwood J, Dooley J, Scott A, Joiner R. Constantly connected. The effects of smart-devices on mental health. Computers in Human Behavior. 2014; 34:267–272. 10.1016/j.chb.2014.02.006

[19] Abi-Jaoude E, Naylor KT, Pignatiello A. Smartphones, social media use and youth mental health. CMAJ. 2020;192(6):E136–E141. doi: 10.1503/cmaj.190434 32041697 PMC7012622

[20] Winther D K. How Does the Time Children Spend Using Digital Technology Impact their Mental Well-being, Social Relationships and Physical Activity? An Evidence-focused literature review. 2017. UNICEF Office of Research–Innocenti. Florence. https://www.unicef-irc.org/publications/pdf/Children-digital-technology-wellbeing.pdf

[21] Vandewater EA, Denis LM. Media, Social Networking, and Pediatric Obesity. Pediatric clinics of North America. 2011;58(6):1509–xii. doi: 10.1016/j.pcl.2011.09.012 22093866 PMC5737742

[22] Greitemeyer T, Mügge D. Video games do affect social outcomes: A meta-analytic review of the effects of violent and prosocial video game play. Personality and Social Psychology Bulletin. 2014; 40 (5): 578–89. doi: 10.1177/0146167213520459 24458215

[23] Radovic A, McCarty CA, Katzman K, Richardson LP. Adolescents’ Perspectives on Using Technology for Health: Qualitative Study. JMIR pediatrics and parenting. 2019; 1(1), e2. 10.2196/pediatrics.8677 PMC6364836 30740590

[24] Kwon M, Kim DJ, Cho H, Yang S. The smartphone addiction scale: development and validation of a short version for adolescents. PloS one. 2013; 8(12):e83558. doi: 10.1371/journal.pone.0083558 24391787 PMC3877074

[25] Soni R, Upadhyay R, Jain M. Prevalence of smart phone addiction, sleep quality and associated behaviour problems in adolescents. International Journal of Research in Medical Sciences. 2017; 5(2): 515–519. 10.18203/2320-6012.ijrms20170142

[26] Canadian Paediatric Society Digital Health Task Force, Ottawa Ontario. Digital media: Promoting healthy screen use in school-aged children and adolescents. Paediatr Child Health. 2019;24(6):402‐417. doi: 10.1093/pch/pxz095 31528113 PMC6736327

[27] Domoff SE, Foley RP, Ferkel R. Addictive phone use and academic performance in adolescents. Hum Behavior & Emerging Technologies. 2019; 2:33–38. 10.1002/hbe2.171

[28] Chen YF. The relationship of mobile phone use to addiction and depression amongst American college students. Mobile Communication and Social Change. 2004; 10: 344–52.

[29] Chen B, Liu F, Ding S, Ying X, Wang L, Wen Y. Gender differences in factors associated with smartphone addiction: A cross-sectional study among medical college students. BMC Psychiatry. 2017; 17(1): 341. doi: 10.1186/s12888-017-1503-z 29017482 PMC5634822

[30] Lee SY, Lee D, Nam CR, Kim DY, Park S, Kwon JG, et al. Distinct patterns of Internet and smartphone-related problems among adolescents by gender: Latent class analysis. Journal of behavioral addictions. 2018; 7(2): 454–465. doi: 10.1556/2006.7.2018.28 29788762 PMC6174601

[31] Taywade A, Khubalkar R. Gender differences in smartphone usage patterns of adolescents. The International Journal of Indian Psychology. 2019; 7:509–515. doi: 10.25215/0704.060

[32] Boumosleh JM, Jaalouk D. Depression, anxiety, and smartphone addiction in university students: A cross-sectional study. PLos ONE. 2017;12(8):e0182239. doi: 10.1371/journal.pone.0182239 28777828 PMC5544206

[33] Babadi-Akashe Z, Zamani BE, Abedini Y, Akbari H, Hedayati N. The Relationship between Mental Health and Addiction to Mobile Phones among University Students of Shahrekord, Iran. Addiction & Health. 2014; 6(3–4): 93–99. 25984275 PMC4354213

[34] Yang G, Tan GX, Li YX, Liu HY, Wang ST. Physical Exercise Decreases the Mobile Phone Dependence of University Students in China: The Mediating Role of Self-Control. International Journal of Environmental Research and Public Health. 2019; 16(21): 4098. doi: 10.3390/ijerph16214098 31652978 PMC6862431

[35] Samaha M, Hawi N. Relationships among smartphones addiction, stress, academic performance, and satisfaction with life. Computers in Human Behavior. 2016; 57: 321–325.

[36] Lepp A., Barkley J., & Karpinski A. (2014). The relationship between cell phone use, academic performance, anxiety, and satisfaction with life in college students. Computers in Human Behavior, 31, 343–350. doi: 10.1016/j.chb.2013.10.049

[37] Hatch E. Determining the Effects of Technology on Children. Senior honors projects. The University of Rhode Island; 2011.

[38] Nwankwo F, Shin HD, Al-Habaibeh A, Massoud H. Evaluation of Children’s Screen Viewing Time and Parental Role in Household Context. Global Pediatrics Health. 2019; 22: 6:2333794X19878062. doi: 10.1177/2333794X19878062 31579685 PMC6757492

[39] Soltani PR, Ghanbari A, Rad AH. Obesity related factors in school-aged children. Iran. J. Nurs. Midwifery Research. 2013; 18: 75–179. PMC3748533 23983750

[40] Zou Y, Xia N, Zou Y, Chen Z, Wen Y. Smartphone addiction may be associated with adolescent hypertension: A cross-sectional study among junior school students in China. BMC Pediatrics. 2019; 19(1): 310. doi: 10.1186/s12887-019-1699-9 31484568 PMC6724312

