# Supplementary material for: The associations between smart device use and psychological distress among secondary and high school students in Kuwait
Source: PLoS One. 2021 Jun 15;16(6):e0251479. doi: 10.1371/journal.pone.0251479 (PMC8205156; doi:10.1371/journal.pone.0251479)
Supplement: S1 File — (DOCX) [file pone.0251479.s001.docx]

1. **قسم الإجتماعي والدموغرافي**

**الرجاء اختيار الاجابة المناسبة كل من الاتي:**

1. **الجنس:**

**🞏** ذكر **🞏** أنثى

1. **العمر :** ___________ سنوات
2. **الجنسية :**

🞏 كويتي 🞏غير كويتي

1. **المستوى التعليمي:**

🞏 المرحلة المتوسطة **……** 🞏الثانوية ……..

1. **التقدير العام للفصل الدراسي الأخير في التقرير:**

🞏 90 – 100 % 🞏 80 – 89 %

🞏 70 – 79 % 🞏 60 – 69 %

🞏 أقل من 60 %

1. **المنطقة التعليمية:**

🞏 العاصمة 🞏 الفروانية

🞏 حولي 🞏 الجهراء

🞏 مبارك الكبير 🞏 الاحمدي

1. **الخصائص البدنية للطالب :**
2. **الوزن ___________ كغ الطول ___________ سم**
3. **هل تمارس أي نشاط رياضي بشكل منتظم (ثلاث مرات أو أكثر في الأسبوع)؟**

🞏 نعم

🞏 لا

**ت-نمط** **استخدام الأجهزة الذكية**

1. **متوسط مجموع ساعات استخدام الأجهزة الذكية يوميًا:**

🞏 أقل من ساعتين 🞏 ساعتان – 4 ساعات 🞏 أكثر من 4 ساعات

1. **متوسط الوقت المستغرق لك على شاشة الجهاز الذكي لكل جلسة:**

🞏 أقل من ساعة واحدة

🞏 من ساعة واحدة الى ساعتين 🞏 أكثر من ساعتين

- **مقياس الاستخدام المفرط للأجهزة الذكية :**

1. **اضاعة العمل المخطط له بسبب استخدام الأجهزة الذكية.**

🞏 موافق بشدة 🞏 موافق 🞏 غير موافق 🞏 غير موافق بشدة

1. **وجود صعوبة في التركيز في الفصل،أو أثناء أداء المهام بسبب استخدام الأجهزة الذكية.**

🞏 موافق بشدة 🞏 موافق 🞏 غير موافق 🞏 غير موافق بشدة

1. **الشعور بالألم في المعصمين أو في الجزء الخلفي من الرقبة أثناء استخدام الأجهزة الذكية.**

🞏 موافق بشدة 🞏 موافق 🞏 غير موافق 🞏 غير موافق بشدة

1. **عدم امكانية الإستغناء عن استخدام الأجهزة الذكية.**

🞏 موافق بشدة 🞏 موافق 🞏 غير موافق 🞏 غير موافق بشدة

1. **الشعور بنفاد الصبر و الضيق عندما لا استخدم الأجهزة الذكية.**

🞏 موافق بشدة 🞏 موافق 🞏 غير موافق 🞏 غير موافق بشدة

1. **وجود الأجهزة الذكية في ذهني حتى عندما لا أستخدمهم.**

🞏 موافق بشدة 🞏 موافق 🞏 غير موافق 🞏 غير موافق بشدة

1. **لن أتخلى عن إستخدام الأجهزة الذكية حتى عندما تكون حياتي اليومية متأثرة بهذا الإستخدام بشكل كبير.**

🞏 موافق بشدة 🞏 موافق 🞏 غير موافق 🞏 غير موافق بشدة

1. **بإستمرار أتحقق من الأجهزة الذكية حتى لا تفوت المحادثات بيني وبين الأشخاص الآخرين على سناب جات و إنستقرام.**

🞏 موافق بشدة 🞏 موافق 🞏 غير موافق 🞏 غير موافق بشدة

1. **استخدام الأجهزة الذكية لفترة أطول مما كنت أنوي.**

🞏 موافق بشدة 🞏 موافق 🞏 غير موافق 🞏 غير موافق بشدة

1. **الناس من حولي يقولون لي إنني أستخدم هاتفي الأجهزة الذكية أكثر من اللازم.**

🞏 موافق بشدة 🞏 موافق 🞏 غير موافق 🞏 غير موافق بشدة

- **مقياس الإكتئاب والقلق والإجهاد - 21 منتجًا (DASS-21)**

يرجى قراءة كل عبارة ووضع دائرة حول الرقم 0 أو 1 أو 2 أو 3 التي تشير إلى مقدار البيان المطبق عليك. لا توجد اجابات صحيحة أو خاطئة، واختر إجابتك بسهولة، مستخدما التقييم التالي:

**مقياس التقييم هو كما يلي:**

**0** لا ينطبق علي نهائيا

**1** ينطبق عليّ إلى درجة ما، أو بعض الأحيان

**2** ينطبق عليّ بدرجة كبيرة أو كثيرا من الأحيان

**3** يطبق عليّ كثيراً

1. **(ت) لقد وجدت صعوبه في التقليل من استخدامه 0 1 2 3**
2. **(ق) اعاني بجفاف بفمي 0 1 2 3**
3. **(إ) لم أستطع أن أواجه أي شعور إيجابي على الاطلاق 0 1 2 3**
4. **(ق) عانيت من صعوبة في التنفس (مثل التنفس السريع بشكل مفرط وضيق التنفس في غياب المجهود البدني)**

**0 1 2 3**

1. **(إ) لقد وجدت صعوبة في العمل للمبادرة بإنجاز المهام 0 1 2 3**
2. **(ت) بدأت أميل إلى المبالغة في ردة الفعل 0 1 2 3**
3. **(ق) لقد شعرت بالارتجاف (على سبيل المثال في اليدين) 0 1 2 3**
4. **(ت) شعرت أنني كنت أستخدم الكثير من الطاقة العصبية 0 1 2 3**
5. **(ق) كنت قلقا من المواقف التي قد أشعر فيها بالذعر والإحباط من نفسي**

**0 1 2 3**

1. **(إ) شعرت أنه ليس لدي ما أتطلع إليه 0 1 2 3**
2. **(ت) وجدت نفسي مضطربا 0 1 2 3**
3. **(ت) وجدت صعوبة في الإسترخاء 0 1 2 3**
4. **(إ) شعرت بانخفاض دقات القلب 0 1 2 3**
5. **(ت) كنت غير متسامح/ة مع أي شيء يمنعني من الاستمرار في ما كنت أفعله**

**0 1 2 3**

1. **(ق) شعرت بانني كنت على وشك الهلع 0 1 2 3**
2. **(إ) لم أكن قادراً على التحمس لأي شيء 0 1 2 3**
3. **(إ) شعرت إنني لا أقدرعلى أن أكون شخصًا ذا أهمية 0 1 2 3**
4. **(ت) شعرت بإنني كنت حساساً 0 1 2 3**
5. **(ق) كنت على بينة من إجهاد قلبي في غياب المجهود البدني (على سبيل المثال بمعنى زيادة**

**معدل ضربات القلب، خفقان القلب)**

**0 1 2 3**

1. **(ق) شعرت بالخوف من دون أي سبب وجيه 0 1 2 3**
2. **(إ) شعرت أن الحياة لا معنى لها 0 1 2 3**

**شكرا جزيلا لمشاركتك !**
