# Supplementary material for: The associations between smart device use and psychological distress among secondary and high school students in Kuwait
Source: PLoS One. 2021 Jun 15;16(6):e0251479. doi: 10.1371/journal.pone.0251479 (PMC8205156; doi:10.1371/journal.pone.0251479)
Supplement: S2 File — (DOCX) [file pone.0251479.s002.docx]

**Section 1: Socio-demographics**

**1. Gender:**

⬜_1_ Male ⬜_2_ Female

**2. Age:** ___________ years

**3. Nationality:**

⬜_1_ Kuwaiti ⬜_2_ Non-Kuwaiti

**4. Educational level:**

⬜_1_ Secondary...……. ⬜_2_ High school……….

**5. Last semester’s overall grade in report:**

⬜_1_ A (90–100%) ⬜_2_ B (80–89%)

⬜_3_ C (70–79%) ⬜_4_ D (60–69%)

⬜_5_ F (less than 60%)

**6**. **Educational region:**

⬜_1_ Al-Farwaniyah ⬜_2_ Hawally

⬜_3_ Al-Asimah ⬜_4_ Al-Jahra

⬜_5_ Mubarak Al-Kabeer ⬜_6_ Al-Ahmadi

**Section 2: Student’s physical attributes**

**7. Weight:** ___________ kg **Height** ___________ **Cm**

**8. Do you play any sport on a regular basis (three or more times per week)?**

⬜_1_ Yes

⬜_2_ No

**Section 3: Student’s pattern of SD use**

**9. Average total hours of smart device use per day:**

⬜_1_ Less than 2 hours ⬜_2_ 2–4 hours ⬜_3_ More than 4 hours

**10. Average screen time spent on the smart device per session:**

⬜_1_ Less than 1 hour ⬜_2_ 1 hour- 2 hours ⬜_3_ More than 2 hours

**E. Psychological-related problems**

**I. Smartphone addiction scale (SAS)-Short Version (10 items)**

This scale is provided with a four-point Likert scale (1 = strongly agree, 2 = agree, 3 = disagree, 4 = strongly disagree). Please read each statement and choose the appropriate response to you

1. Missing planned work due to smartphone use.  **1** **2** **3** **4**
2. Having a hard time concentrating in class, while doing assignments, or while working due to smartphone use.

**1** **2** **3** **4**

1. Feeling pain in the wrists or at the back of the neck while using a smartphone. **1** **2** **3** **4**
2. Won’t be able to stand not having a smartphone.  **1** **2** **3** **4**
3. Feeling impatient and fretful when I am not holding my smartphone. **1** **2** **3** **4**
4. Having my smartphone in my mind even when I am not using it.  **1** **2** **3** **4**
5. I will never give up using my smartphone even when my daily life is already greatly affected by.

**1** **2** **3** **4**

1. Constantly checking my smartphone so as not to miss conversations between other people on Snapchat or Instagram.

**1** **2** **3** **4**

1. Using my smartphone longer than I had intended.  **1** **2** **3** **4**
2. The people around me tell me that I use my smartphone too much. **1** **2** **3** **4**

**II. Depression, Anxiety and Stress Scale - 21 Items (DASS-21)**

The scale is provided with a four-point Likert frequency scale (0 = never applies to me, 1 = sometimes applies to me, 2 = often applies to me, 3 = always applies to me)

Please read each statement and circle a number **0**, **1**, **2** or **3** which indicates how much the statement applied to you over the past week. There are no right or wrong answers. Do not spend too much time on any statement.

1 (s) I found it hard to wind down **0** **1** **2** **3**

2 (a) I was aware of dryness of my mouth **0** **1** **2** **3**

3 (d) I couldn’t seem to experience any positive feeling at all **0** **1** **2** **3**

4 (a) I experienced breathing difficulty (e.g. excessively rapid breathing, breathlessness in the absence of physical exertion)

**0** **1** **2** **3**

5 (d) I found it difficult to work up the initiative to do things **0** **1** **2** **3**

6 (s) I tended to over-react to situations **0** **1** **2** **3**

7 (a) I experienced trembling (e.g. in the hands) **0** **1** **2** **3**

8 (s) I felt that I was using a lot of nervous energy **0** **1** **2** **3**

9 (a) I was worried about situations in which I might panic and make a fool of myself **0** **1** **2** **3**

10 (d) I felt that I had nothing to look forward to **0** **1** **2** **3**

11 (s) I found myself getting agitated **0** **1** **2** **3**

12 (s) I found it difficult to relax **0** **1** **2** **3**

13 (d) I felt down-hearted and blue **0** **1** **2** **3**

14 (s) I was intolerant of anything that kept me from getting on with what I was doing **0** **1** **2** **3**

15 (a) I felt I was close to panic **0** **1** **2** **3**

16 (d) I was unable to become enthusiastic about anything **0** **1** **2** **3**

17 (d) I felt I wasn’t worth much as a person **0** **1** **2** **3**

18 (s) I felt that I was rather touchy **0** **1** **2** **3**

19 (a) I was aware of the action of my heart in the absence of physical exertion (e.g. sense of heart rate increase, heart missing a beat) **0** **1** **2** **3**

20 (a) I felt scared without any good reason **0** **1** **2** **3**

21 (d) I felt that life was meaningless **0** **1** **2** **3**

**Thank you very much for your participation**
